# Checklist of the superfamilies Oestroidea and Hippoboscoidea of Finland (Insecta, Diptera)

**DOI:** 10.3897/zookeys.441.7252

**Published:** 2014-09-19

**Authors:** Jaakko Pohjoismäki, Jere Kahanpää

**Affiliations:** 1 University of Eastern Finland, Department of Biology, P.O.Box 111, FI-80101 Joensuu, Finland; 2 Finnish Museum of Natural History, Zoology Unit, P.O. Box 17, FI-00014 University of Helsinki, Finland

**Keywords:** Checklist, Finland, Diptera, Oestroidea, Hippoboscoidea

## Abstract

An updated checklist of the superfamilies Oestroidea and Hippoboscoidea recorded from Finland is presented. The checklist covers the following families: Calliphoridae, Rhiniidae, Sarcophagidae, Rhinophoridae, Tachinidae, Oestridae and Hippoboscidae.

## Introduction

Oestroidea is a large and diverse superfamily of flies with a variety of life history strategies ranging from saprophages to parasitoids or endoparasites, sometimes even within the family. Some groups, such as certain subfamilies of the Calliphoridae, have also significant veterinary, medical and economical impact. Oestroidea are regarded as monophyletic clade, however the same does not apply to the family of Calliphoridae within Oestroidea (Marinho et al. 2012). Besides Calliphoridae, Oestroidea currently includes six other monophyletic families: Rhiniidae, Sarcophagidae, Rhinophoridae, Tachinidae, Oestridae and the non-finnish Mystacinobiidae. Additionally, the subfamilies of Oestridae (Oestrinae, Gasterophilinae) are sometimes treated as full families.

Hippoboscoidea contains the louse flies Hippoboscidae as well as the tropical Glossinidae or tsetse flies. Hippoboscidae is divided to the mammal or bird parasitic louse flies (Hippoboscinae) and batflies (Nycterbiinae, Streblinae). Although batflies have been treated as full families, they seem to be subordinate to the other Hippoboscidae (Kutty et al. 2010). The checklist will therefore follow the family division of Pape et al. 2011.

While world catalogues has been published for Sarcophagidae (Pape 1996) and Hippoboscidae (Dick 2006) only regional (Herting and Dely-Draskovits 1993) or genus-level (O’Hara 2012) catalogues exist for the other oestroid and hippoboscoid families. The Finnish species of Oestroidea and Hippoboscoidea were last listed by Hackman (1980). The sarcophagid checklist was updated by Pape (1987a,b) and the calliphorids by Rognes (1991). The updated checklists for Oestridae, Rhinophoridae and Tachinidae follow the taxonomic order of Pape (2001), Herting (1993) and Herting and Dely-Draskovits (1993), respectively.

**Table 1.** Number of species by family.

Family	Number of species in			Level of knowledge
	World (Pape et al. 2011)	Europe	Finland	
**Oestroidea:**				
Calliphoridae	1525	102	45	good
Rhiniidae	376	12	0–1	good
Sarcophagidae	3094	309	64	average
Rhinophoridae	174	45	5	average
Tachinidae	9626	877	319	average
Oestridae	176	22	8	average
**Hippoboscoidea:**				
Hippoboscidae s. lat.	782	45	12	average

## Checklist part 1: Oestroidea

suborder Brachycera Macquart, 1834

clade Eremoneura Lameere, 1906

clade Cyclorrhapha Brauer, 1863

infraorder Schizophora Becher, 1882

clade Muscaria Enderlein, 1936

parvorder Calyptratae Robineau-Desvoidy, 1830

superfamily Oestroidea Leach, 1815

**CALLIPHORIDAE** Brauer & Bergenstamm, 1889

CALLIPHORINAE Brauer & Bergenstamm, 1889

***BELLARDIA*** Robineau-Desvoidy, 1863

= ***Pseudonesia*** Villeneuve, 1920

*Bellardia bayeri* (Jacentkovsky, 1937)

*Bellardia pandia* (Walker, 1849)

= *biseta* (Kramer, 1917)

*Bellardia pubicornis* (Zetterstedt, 1838)

= *puberula* (Zetterstedt, 1838)

*Bellardia stricta* (Villeneuve, 1926)

= *polita* auct. nec (Mik, 1883)

*Bellardia viarum* (Robineau-Desvoidy, 1830)

= *pusilla* (Meigen, 1826)

*Bellardia vulgaris* (Robineau-Desvoidy, 1830)

= *agilis* (Meigen, 1826) preocc.

***CALLIPHORA*** Robineau-Desvoidy, 1830

= ***Steringomyia*** Pokorny, 1889

= ***Acrophaga*** Brauer & Bergenstamm, 1891

= ***Abonesia*** Villeneuve, 1927

= ***Acronesia*** Hall, 1948

*Calliphora genarum* (Zetterstedt, 1838)

= *alpina* (Zetterstedt, 1845)

*Calliphora loewi* Enderlein, 1903

*Calliphora stelviana* (Brauer & Bergenstamm, 1891)

*Calliphora subalpina* (Ringdahl, 1931)

*Calliphora uralensis* Villeneuve, 1922

*Calliphora vicina* Robineau-Desvoidy, 1830

= *erythrocephala* (Meigen, 1826) preocc.

*Calliphora vomitoria* (Linnaeus, 1758)

***CYNOMYA*** Robineau-Desvoidy, 1830

*Cynomya mortuorum* (Linnaeus, 1761)

***ONESIA*** Robineau-Desvoidy, 1830

*Onesia floralis* Robineau-Desvoidy, 1830

CHRYSOMYINAE Shannon, 1923

***PROTOCALLIPHORA*** Hough, 1899

*Protocalliphora azurea* (Fallén, 1817)

*Protocalliphora nuortevai* Grunin, 1972

*Protocalliphora peusi* Gregor & Povolny, 1959

*Protocalliphora proxima* Grunin, 1966

*Protocalliphora rognesi* Thompson & Pont, 1993

= *chrysorrhoea* Meigen, 1826 preocc.

***PROTOPHORMIA*** Townsend, 1908

= ***Boreellus*** Aldrich & Schannon, 1923

*Protophormia atriceps* (Zetterstedt, 1845)

*Protophormia terraenovae* (Robineau-Desvoidy, 1830)

***TRYPOCALLIPHORA*** Peus, 1960

*Trypocalliphora braueri* (Hendel, 1901)

= *lindneri* Peus, 1960

HELICOBOSCINAE Verves, 1980

***EURYCHAETA*** Brauer & Bergenstamm, 1891

= ***Helicobosca*** Bezzi, 1906

*Eurychaeta palpalis* (Robineau-Desvoidy, 1830)

LUCILIINAE Shannon, 1923

***LUCILIA*** Robineau-Desvoidy, 1830

*Lucilia ampullacea* Villeneuve, 1922

*Lucilia bufonivora* Moniez, 1876

*Lucilia caesar* (Linnaeus, 1758)

*Lucilia illustris* (Meigen, 1826)

*Lucilia magnicornis* (Siebke, 1863)

= *fuscipalpis* (Zetterstedt, 1845)

*Lucilia richardsi* Collin, 1926

*Lucilia sericata* (Meigen, 1826)

*Lucilia silvarum* (Meigen, 1826)

MELANOMYINAE Townsend, 1919

***ANGIONEURA*** Brauer & Bergenstamm, 1893

*Angioneura acerba* (Meigen, 1838)

***MELANOMYA*** Rondani, 1856

*Melanomya nana* (Meigen, 1826)

***MELINDA*** Robineau-Desvoidy, 1830

*Melinda gentilis* Robineau-Desvoidy, 1830

= *coerulea* (Meigen, 1826) preocc.

= *cognata* (Meigen, 1830)

*Melinda viridicyanea* (Robineau-Desvoidy, 1830)

POLLENIINAE Brauer & Bergenstamm, 1889

***MORINIA*** Robineau-Desvoidy, 1830

*Morinia doronici* (Scopoli, 1763)

= *melanoptera* (Fallén, 1817)

= *Angioneura fimbriata* misid.

***POLLENIA*** Robineau-Desvoidy, 1830

*Pollenia amentaria* (Scopoli, 1763)

= *vespillo* auct. nec (Fabricius, 1794)

*Pollenia angustigena* Wainwright, 1940

*Pollenia griseotomentosa* (Jacentkowský, 1944)

*Pollenia hungarica* Rognes, 1987

*Pollenia labialis* Robineau-Desvoidy, 1863

= *excarinata* Wainwright, 1940

= *intermedia* misid.

*Pollenia pediculata* Macquart, 1834

*Pollenia rudis* (Fabricius, 1794)

= *varia* (Meigen, 1826)

*Pollenia vagabunda* (Meigen, 1826)

**RHINIIDAE** Brauer & Bergenstamm, 1889

***STOMORHINA*** Rondani, 1861

*Stomorhina lunata* (Fabricius, 1805)

**SARCOPHAGIDAE** Macquart, 1834

MILTOGRAMMINAE Lioy, 1864

***AMOBIA*** Robineau-Desvoidy, 1830

*Amobia oculata* (Zetterstedt, 1844)

= *signata* misid.

*Amobia signata* (Meigen, 1824)

***HILARELLA*** Rondani, 1856

*Hilarella hilarella* (Zetterstedt, 1844)

***MACRONYCHIA*** Rondani, 1859

= ***Macronichia*** orig. emend.

**sg. *Macronychia*** Rondani, 1859

*Macronychia striginervis* (Zetterstedt, 1838)

**sg. *Moschusa*** Robineau-Desvoidy, 1863

*Macronychia agrestis* (Fallén, 1820)

*Macronychia griseola* (Fallén, 1820)

*Macronychia polyodon* (Meigen, 1824)

***METOPIA*** Meigen, 1803

*Metopia argentata* Macquart 1850

= *roserii* Rondani, 1859

= *stackelbergi* Rohdendorf, 1955

*Metopia argyrocephala* (Meigen, 1824)

= *leucocephala* (Rossi, 1790) preocc.

*Metopia campestris* (Fallén, 1810)

*Metopia grandii* Venturi, 1953

*Metopia staegerii* Rondani, 1859

= *rondaniana* Venturi, 1953

*Metopia tshernovae* Rohdendorf, 1955

***MILTOGRAMMA*** Meigen, 1803

*Miltogramma germari* Meigen, 1824

*Miltogramma iberica* (Villeneuve, 1912)

*Miltogramma oestracea* (Fallén, 1820)

*Miltogramma punctata* Meigen, 1824

*Miltogramma villeneuvei* Verves, 1982

***OEBALIA*** Robineau-Desvoidy, 1863

*Oebalia cylindrica* (Fallén, 1810)

*Oebalia minuta* (Fallén, 1810)

***PHROSINELLA*** Robineau-Desvoidy, 1863

*Phrosinella sannio* (Zetterstedt, 1838)

= *nasuta* misid.

***PTERELLA*** Robineau-Desvoidy, 1830

*Pterella grisea* (Meigen, 1824)

***SENOTAINIA*** Macquart, 1846

*Senotainia albifrons* (Rondani, 1859)

*Senotainia conica* (Fallén, 1810)

*Senotainia puncticornis* (Zetterstedt, 1859)

= *imberbis* (Zetterstedt, 1838) preocc.

= *crabronum* (Kramer, 1920)

***TAXIGRAMMA*** Perris, 1852

*Taxigramma elegantulum* (Zetterstedt, 1844)

*Taxigramma heteroneurum* (Meigen, 1830)

PARAMACRONYCHIINAE Brauer & Bergenstamm, 1889

***AGRIA*** Robineau-Desvoidy, 1830

*Agria affinis* (Fallén, 1817)

= *punctata* Robineau-Desvoidy, 1830

*Agria mamillata* (Pandellé, 1896)

***ANGIOMETOPA*** Brauer & Bergenstamm, 1889

*Angiometopa falleni* Pape, 1986

= *ruralis* (Fallén, 1817) preocc.

***BRACHICOMA*** Rondani, 1856

*Brachicoma devia* (Fallén, 1820)

*Brachicoma borealis* Ringdahl, 1932

SARCOPHAGINAE Macquart, 1834

***BLAESOXIPHA*** Loew, 1861

**sg. *Blaesoxipha*** Loew, 1861

*Blaesoxipha lapidosa* Pape, 1994

= *agrestis* auct. nec (Robineau-Desvoidy, 1863)

= *campestris* auct. nec (Robineau-Desvoidy, 1863)

*Blaesoxipha laticornis* (Meigen, 1826)

*Blaesoxipha plumicornis* (Zetterstedt, 1859)

= *laticornis* misid.

= *gladiatrix* (Pandellé, 1896)

**sg. *Servaisia*** Robineau-Desvoidy, 1863

*Blaesoxipha erythrura* (Meigen, 1826)

***RAVINIA*** Robineau-Desvoidy, 1830

*Ravinia pernix* (Harris, 1780)

= *striata* (Fabricius, 1794) preocc.

***SARCOPHAGA*** Meigen, 1826

**sg. *Bellieriomima*** Rohdendorf, 1937

*Sarcophaga subulata* Pandellé, 1896

**sg. *Discacheata*** Enderlein, 1928

*Sarcophaga pumila* Meigen, 1826

**sg. *Helicophagella*** Enderlein, 1928

*Sarcophaga crassimargo* Pandellé, 1896

*Sarcophaga melanura* Meigen, 1826

*Sarcophaga rosellei* Böttcher, 1912

**sg. *Heteronychia*** Brauer & Bergenstamm, 1889

*Sarcophaga depressifrons* Zetterstedt, 1845

= *offuscata* auct. nec Meigen, 1826

*Sarcophaga haemorrhoa* Meigen, 1826

*Sarcophaga proxima* (Rondani, 1860)

*Sarcophaga vagans* (Meigen, 1826)

= *frenata* Pandellé, 1896

*Sarcophaga vicina* (Macquart, 1835)

**sg. *Liosarcophaga*** Enderlein, 1928

*Sarcophaga emdeni* (Rohdendorff, 1969)

= *teretirostris* auct. nec Pandellé, 1896

*Sarcophaga portschinskyi* Rohdendorf, 1937

= *tuberosa* misid.

**sg. *Mehria*** Enderlein, 1928

*Sarcophaga sexpunctata* (Fabricius, 1805)

= *clathrata* (Meigen, 1826)

*Sarcophaga nemoralis* Kramer, 1908

**sg. *Myorhina*** Robineau-Desvoidy, 1830

= ***Pierretia*** Robineau-Desvoidy, 1863

*Sarcophaga socrus* Rondani, 1860

= *rostrata* Pandellé, 1896

*Sarcophaga villeneuvei* Böttcher, 1912

**sg. *Pandelleisca*** Rohdendorf, 1937

*Sarcophaga similis* Meade, 1876

**sg. *Parasarcophaga*** Johnston & Tiegs, 1921

*Sarcophaga albiceps* Meigen, 1826

= *privigna* Rondani, 1860

**sg. *Robineauella*** Enderlein, 1928

*Sarcophaga caerulescens* Zetterstedt, 1838

= *scoparia* Pandellé, 1896

**sg. *Rosellea*** Rohdendorf, 1937

*Sarcophaga aratrix* Pandellé, 1896

**sg. *Sarcophaga*** Meigen, 1826

*Sarcophaga carnaria* (Linnaeus, 1758)

= *schulzi* Müller, 1922

= *lehmanni* auct. nec Müller, 1922

*Sarcophaga lehmanni* Müller, 1922

= *lasiostyla* auct. nec Macquart, 1843

= *cognata* Rondani, 1860 preocc.

*Sarcophaga subvicina* Rohdendorf, 1937

*Sarcophaga variegata* (Scopoli, 1763)

= *carnaria* auct. nec (Linnaeus, 1758)

**sg. *Sarcotachinella*** Townsend, 1892

*Sarcophaga sinuata* Meigen, 1826

**sg. *Thyrsocnema*** Enderlein, 1928

*Sarcophaga incisilobata* Pandellé, 1896

*Sarcophaga kentejana* (Rohdendorf, 1937)

= *lapponica* (Tiensuu, 1939)

**RHINOPHORIDAE** Robineau-Desvoidy, 1863

***MELANOPHORA*** Meigen, 1803

*Melanophora roralis* (Linnaeus, 1758)

***PAYKULLIA*** Robineau-Desvoidy, 1830

*Paykullia brevicornis* (Zetterstedt, 1844)

*Paykullia maculata* (Fallén, 1815)

***STEVENIA*** Robineau-Desvoidy, 1830

*Stevenia atramentaria* (Meigen, 1824)

***TRICOGENA*** Rondani, 1856

= ***Frauenfeldia*** Egger, 1865

*Tricogena rubricosa* (Meigen, 1824)

**TACHINIDAE** Robineau-Desvoidy, 1830

EXORISTINAE Robineau-Desvoidy, 1863

tribe Exoristini Robineau-Desvoidy, 1863

***EXORISTA*** Meigen, 1803

**sg. *Adenia*** Robineau-Desvoidy, 1863

*Exorista mimula* (Meigen, 1824)

*Exorista rustica* (Fallén, 1810)

*Exorista* sp. A

**sg. *Exorista*** Meigen, 1803

*Exorista fasciata* (Fallén, 1820)

*Exorista larvarum* (Linnaeus, 1758)

**sg. *Podotachina*** Brauer & Bergenstamm, 1891

*Exorista grandis* (Zetterstedt, 1844)

= *sorbillans* auct. nec Wiedemann, 1830

**sg. *Ptilotachina*** Brauer & Bergenstamm 1891

*Exorista deligata* Pandellé, 1896

***CHETOGENA*** Rondani, 1856

*Chetogena tschorsnigi* Ziegler, 1999

***DIPLOSTICHUS*** Brauer & Bergenstamm, 1889

*Diplostichus janitrix* (Hartig, 1838)

***PHOROCERA*** Brauer & Bergenstamm, 1889

*Phorocera assimilis* (Fallén, 1810)

*Phorocera obscura* (Fallén, 1810)

***PHORINIA*** Robineau-Desvoidy, 1830

*Phorinia aurifrons* Robineau-Desvoidy, 1830

***BESSA*** Robineau-Desvoidy, 1830

*Bessa selecta* (Meigen, 1824)

tribe Blondeliini Robineau-Desvoidy, 1863

***BELIDA*** Robineau-Desvoidy, 1863

= ***Aporotachina*** Meade, 1894

*Belida angelicae* (Meigen, 1824)

***MEIGENIA*** Robineau-Desvoidy, 1830

*Meigenia dorsalis* (Meigen, 1824)

*Meigenia mutabilis* (Fallén, 1810)

= *bisignata* (Meigen, 1824)

***ZAIRA*** Robineau-Desvoidy, 1830

= ***Viviania*** Rondani, 1861

*Zaira cinerea* (Fallén, 1810)

***MEDINA*** Robineau-Desvoidy, 1830

*Medina collaris* (Fallén, 1820)

*Medina luctuosa* (Meigen, 1824)

*Medina separata* (Meigen, 1824)

***POLICHETA*** Rondani, 1856

= ***Perichaeta*** preocc.

*Policheta unicolor* (Fallén, 1820)

***ISTOCHETA*** Rondany, 1859

= ***Hyperecteina*** Schiner, 1862

*Istocheta longicornis* (Fallén, 1810)

***STAUROCHAETA*** Brauer & Bergenstamm, 1889

*Staurochaeta albocingulata* (Fallén, 1820)

***ADMONTIA*** Brauer & Bergenstamm, 1889

= ***Trichopareia*** Brauer & Bergenstamm, 1889

*Admontia blanda* (Fallén, 1820)

*Admontia grandicornis* (Zetterstedt, 1849)

*Admontia seria* (Meigen, 1824)

***OSWALDIA*** Robineau-Desvoidy, 1863

*Oswaldia muscaria* (Fallén, 1810)

*Oswaldia eggeri* (Brauer & Bergenstamm, 1889)

*Oswaldia reducta* (Villeneuve, 1930)

*Oswaldia spectabilis* (Meigen, 1824)

= *albisquama* (Zetterstedt, 1844)

***PARACRASPEDOTHRIX*** Villeneuve, 1919

*Paracraspedothrix montivaga* Villeneuve, 1919

***LIGERIA*** Robineau-Desvoidy, 1863

*Ligeria angusticornis* (Loew, 1847)

= *zetterstedti* (Ringdahl, 1945)

***LIGERIELLA*** Mesnil, 1961

*Ligeriella aristata* (Villeneuce, 1911)

***BLONDELIA*** Robineau-Desvoidy, 1830

*Blondelia nigripes* (Fallén, 1810)

tribe Acemyini Brauer & Bergenstamm, 1889

***ACEMYA*** Robineau-Desvoidy, 1830

*Acemya acuticornis* (Meigen, 1824)

*Acemya rufitibia* (von Roser, 1840)

tribe Ethillini Mesnil, 1944

***PARATRYPHERA*** Brauer & Bergenstamm, 1891

*Paratryphera barbatula* (Rondani, 1859)

*Paratryphera bisetosa* (Brauer & Bergenstamm, 1891)

tribe Winthemiini Townsend, 1913

***RHAPHIOCHAETA*** Brauer & Bergenstamm, 1889

*Rhaphiochaeta breviseta* (Zetterstedt, 1838)

***SMIDTIA*** Robineau-Desvoidy, 1830

= ***Timavia*** Robineau-Desvoidy, 1863

*Smidtia amoena* (Meigen, 1824)

*Smidtia conspersa* (Meigen, 1824)

***WINTHEMIA*** Robineau-Desvoidy, 1830

*Winthemia cruentata* (Rondani, 1859)

*Winthemia erythrura* (Meigen, 1838)

*Winthemia quadripustulata* (Fabricius, 1794)

*Winthemia venusta* (Meigne, 1824)

***NEMORILLA*** Rondani, 1856

*Nemorilla floralis* (Fallen, 1810)

*Nemorilla maculosa* (Meigen, 1824)

= *floralis* misid.

tribe Eryciini Robineau-Desvoidy, 1830

***APLOMYA*** Robineau-Desvoidy, 1830

*Aplomya confinis* (Fallén, 1820)

***PHEBELLIA*** Robineau-Desvoidy, 1846

*Phebellia clavellariae* (Brauer & Bergenstamm, 1891)

*Phebellia glauca* (Meigen, 1824)

*Phebellia glaucoides* Herting, 1961

*Phebellia glirina* (Rondani, 1859)

*Phebellia margaretae* Bergström, 2005

*Phebellia nigripalpis* (Robineau-Desvoidy, 1847)

= *fuscipennis* (Robineau-Desvoidy, 1830)

*Phebellia pauciseta* (Villeneuve, 1908)

*Phebellia strigifrons* (Zetterstedt, 1838)

*Phebellia stulta* (Zetterstedt, 1844)

*Phebellia triseta* (Pandellé, 1896)

*Phebellia vicina* Wainwright, 1940

*Phebellia villica* (Zetterstedt, 1838)

*Phebellia* sp. A

***NILEA*** Robineau-Desvoidy, 1863

*Nilea hortulana* (Meigen, 1824)

*Nilea innoxia* Robineau-Desvoidy, 1863

*Nilea rufiscutellaris* (Zetterstedt, 1859)

***TLEPHUSA*** Robineau-Desvoidy, 1863

*Tlephusa cincinna* (Meigen, 1859)

= *diligens* auct. nec (Zetterstedt, 1844)

***EPICAMPOCERA*** Macquart, 1849

*Epicampocera succincta* (Meigen, 1824)

***PHRYXE*** Robineau-Desvoidy, 1830

*Phryxe erythrostoma* (Hartig, 1838)

*Phryxe magnicornis* (Zetterstedt, 1838)

*Phryxe nemea* (Meigen, 1824)

*Phryxe vulgaris* (Fallén, 1810)

***PERIARCHICLOPS*** Villeneuve, 1924

*Periarchiclops scutellaris* (Fallén, 1820)

***BACTROMYIA*** Brauer & Bergenstamm, 1891

*Bactromyia aurulenta* (Meigen, 1824)

= *Thecocarcelia acutangula* misid.

***PSEUDOPERICHAETA*** Brauer & Bergenstamm, 1899

*Pseudoperichaeta nigrolineata* (Stephens, 1853)

***LYDELLA*** Robineau-Desvoidy, 1830

*Lydella ripae* (Brischke, 1885)

= *grisescens* misid.

= *lepida* misid.

*Lydella stabulans* (Meigen, 1824)

***CADURCIELLA*** Villeneuve, 1927

*Cadurciella tritaeniata* (Rondani, 1859)

***DRINO*** Robineau-Desvoidy, 1863

*Drino bohemica* Mesnil, 1949

*Drino galii* (Brauer & Bergenstamm, 1891)

*Drino gilva* (Hartig, 1838)

*Drino inconspicua* (Meigen, 1830)

*Drino lota* (Meigen, 1824)

*Drino vicina* (Zetterstedt, 1849)

***HUBNERIA*** Robineau-Desvoidy, 1848

= ***Huebneria*** orig. emend.

*Hubneria affinis* (Fallén, 1810)

***CARCELIA*** Robineau-Desvoidy, 1830

**sg. *Carcelia*** Robineau-Desvoidy, 1830

*Carcelia atricosta* Herting, 1961

= *bombylans* misid.

*Carcelia bombylans* Robineau-Desvoidy, 1830

*Carcelia gnava* (Meigen, 1824)

= *excavata* (Zetterstedt, 1844)

*Carcelia laxifrons* Villeneuve, 1912

*Carcelia lucorum* (Meigen, 1824)

*Carcelia rasa* (Macquart, 1849)

= *amphion* Robineau-Desvoidy, 1863

*Carcelia tibialis* (Robineau-Desvoidy, 1863)

***SENOMETOPIA*** Macquart, 1834

*Senometopia excisa* (Fallén, 1820)

*Senometopia intermedia* (Herting, 1960)

*Senometopia pollinosa* Mesnil, 1941

= *rutilla* auct. nec (Villeneuve, 1912)

= *obesa* auct. nec (Zetterstedt, 1859)

*Senometopia separata* (Rondani, 1859)

= *bombycivora* auct. nec (Robineau-Desvoidy, 1830)

***ERYCIA*** Robineau-Desvoidy, 1830

*Erycia fatua* (Meigen, 1824)

= *festinans* misid.

*Erycia furibunda* (Zetterstedt, 1844)

***XYLOTACHINA*** Brauer & Bergenstamm, 1891

*Xylotachina diluta* (Meigen, 1824)

tribe Goniini Lioy, 1864

***PLATYMYA*** Robineau-Desvoidy, 1830

*Platymya fimbriata* (Meigen, 1824)

***EUMEA*** Robineau-Desvoidy, 1863

= ***Platymya*** Robineau-Desvoidy, 1830 in part

*Eumea linearicornis* (Zetterstedt, 1844)

= *westermanni* (Zetterstedt, 1844)

*Eumea mitis* (Meigen, 1824)

***MYXEXORISTOPS*** Townsend, 1911

*Myxexoristops abietis* Herting, 1964

*Myxexoristops arctica* (Zetterstedt, 1838)

*Myxexoristops bonsdorffi* (Zetterstedt, 1859)

*Myxexoristops stolida* (Stein, 1924)

= *blondeli* misid.

***ZENILLIA*** Robineau-Desvoidy, 1830

*Zenillia fulva* (Fallén, 1820)

= *libatrix* (Panzer, 1798) preocc.

***CLEMELIS*** Robineau-Desvoidy, 1863

*Clemelis pullata* (Meigen, 1824)

***PALES*** Robineau-Desvoidy, 1830

*Pales pavida* (Meigen, 1824)

***CYZENIS*** Robineau-Desvoidy, 1863

*Cyzenis albicans* (Fallén, 1810)

*Cyzenis jucunda* (Meigen, 1838)

***BOTRIA*** Rondani, 1856

= ***Bothria*** orig. emend.

*Botria frontosa* (Meigen, 1824)

*Botria subalpina* Villeneuve, 1910

***CEROMASIA*** Rondani, 1856

*Ceromasia rubrifrons* (Macquart, 1834)

***ALLOPHOROCERA*** Hendel, 1901

= ***Erycilla*** Mesnil, 1957

*Allophorocera ferruginea* (Meigen, 1824)

*Allophorocera lapponica* Wood, 1974

***OCYTATA*** Gistel, 1848

*Ocytata pallipes* (Fallén, 1820)

***ERYNNIA*** Robineau-Desvoidy, 1830

*Erynnia ocypterata* (Fallén, 1810)

= *nitida* (Robineau-Desvoidy, 1830)

***ELODIA*** Robineau-Desvoidy, 1863

*Elodia ambulatoria* (Meigen, 1824)

= *convexifrons* (Zetterstedt, 1844)

***STURMIA*** Robineau-Desvoidy, 1830

*Sturmia bella* (Meigen, 1824)

***BLEPHARIPA*** Rondani, 1856

*Blepharipa pratensis* (Meigen, 1824)

***PROSOPEA*** Rondani, 1861

*Prosopea nigricans* (Egger, 1861)

***HEBIA*** Robineau-Desvoidy, 1830

*Hebia flavipes* Robineau-Desvoidy, 1830

***FRONTINA*** Meigen, 1838

*Frontina laeta* (Meigen, 1824)

***BRACHICHETA*** Rondani, 1861

*Brachicheta strigata* (Meigen, 1824)

***GONIA*** Meigen, 1803

*Gonia capitata* (De Geer, 1776)

*Gonia divisa* Meigen, 1826

*Gonia ornata* Meigen, 1826

*Gonia picea* (Robineau-Desvoidy, 1830)

= *sicula* auct. nec (Robineau-Desvoidy, 1830)

***ONYCHOGONIA*** Brauer & Bergenstamm, 1889

*Onychogonia cervini* (Bigot, 1881)

*Onychogonia flaviceps* (Zetterstedt, 1838)

= *interrupta* (Rondani, 1859)

***SPALLANZANIA*** Robineau-Desvoidy, 1830

*Spallanzania hebes* (Fallén, 1820)

TACHININAE Robineau-Desvoidy, 1830

tribe Polideini Brauer & Bergenstamm, 1889

***LYDINA*** Robineau-Desvoidy, 1830

*Lydina aenea* (Meigen, 1824)

***LYPHA*** Robineau-Desvoidy, 1830

*Lypha dubia* (Fallén, 1810)

*Lypha ruficauda* (Zetterstedt, 1838)

tribe Tachinini Robineau-Desvoidy, 1830

***TACHINA*** Meigen, 1803

= ***Larvaevora*** Meigen, 1800 suppr.

**sg. *Eudoromyia*** Bezzi, 1906

*Tachina fera* (Linnaeus, 1761)

Tachina sp. aff. magnicornis

*Tachina magnicornis* (Zetterstedt, 1844)

**sg. *Servillia*** Robineau-Desvoidy, 1830

*Tachina ursina* (Meigen, 1824)

**sg. *Tachina*** Meigen, 1803

*Tachina grossa* (Linnaeus, 1758)

***NOWICKIA*** Wachtl, 1894

*Nowickia alpina* (Zetterstedt, 1844)

*Nowickia ferox* (Panzer, 1809)

*Nowickia marklini* (Zetterstedt, 1838)

***PELETERIA*** Robineau-Desvoidy, 1830

= ***Cuphocera*** Macquart, 1845

*Peleteria ferina* (Zetterstedt, 1844)

*Peleteria rubescens* Robineau-Desvoidy, 1830

= *nigricornis* (Meigen, 1838)

*Peleteria ruficornis* (Macquart, 1835)

***GERMARIA*** Robineau-Desvoidy, 1830

= ***Atractochaeta*** Brauer & Bergenstamm, 1889

*Germaria angustata* (Zetterstedt, 1844)

*Germaria ruficeps* (Fallén, 1820)

tribe Linnaemyini Townsend, 1919

***LINNAEMYA*** Robineau-Desvoidy, 1830

*Linnaemya haemorrhoidalis* (Fallén, 1810)

*Linnaemya olsufjevi* Zimin, 1954

= *haemorrhoidalis* misid.

*Linnaemya rossica* Zimin, 1954

= *haemorrhoidalis* misid.

*Linnaemya vulpina* (Fallén, 1810)

*Linnaemya* sp. A

***CHRYSOSOMOPSIS*** Townsend, 1916

*Chrysosomopsis aurata* (Fallén, 1820)

***PANZERIA*** Robineau-Desvoidy, 1830

= ***Ernestia*** Robineau-Desvoidy, 1830

*Panzeria puparum* (Fabricius, 1794)

*Panzeria rudis* (Fallén, 1810)

*Panzeria vagans* (Meigen, 1824)

***APPENDICIA*** Stein, 1924

*Appendicia truncata* (Zetterstedt, 1838)

***EURITHIA*** Robineau-Desvoidy, 1844

*Eurithia anthophila* (Robineau-Desvoidy, 1830)

= *radicum* auct. nec Linnaeus, 1758

*Eurithia caesia* (Fallén, 1810)

*Eurithia connivens* (Zetterstedt, 1844)

*Eurithia consobrina* (Meigen, 1824)

*Eurithia intermedia* (Zetterstedt, 1844)

= *conjugata* (Zetterstedt, 1855)

*Eurithia vivida* (Zetterstedt, 1838)

***HYALURGUS*** Brauer & Bergenstamm, 1893

*Hyalurgus crucigerus* (Zetterstedt, 1838)

*Hyalurgus lucidus* (Meigen, 1824)

***NEMORAEA*** Robineau-Desvoidy, 1830

*Nemoraea pellucida* (Meigen, 1824)

***GYMNOCHETA*** Robineau-Desvoidy, 1830

*Gymnocheta magna* (Fallén, 1810)

*Gymnocheta viridis* (Fallén, 1810)

***ZOPHOMYIA*** Macquart, 1835

*Zophomyia temula* (Scopoli, 1763)

***CLEONICE*** Robineau-Desvoidy, 1863

= ***Steiniella*** Berg, 1898 preocc.

*Cleonice callida* (Meigen, 1824)

*Cleonice keteli* Ziegler, 2000

*Cleonice nitidiuscula* (Zetterstedt, 1859)

***LOEWIA*** Egger, 1856

= ***Fortisia*** Rondani, 1861

*Loewia erecta* Bergström, 2007

= *phaeoptera* misid.

*Loewia foeda* (Meigen, 1824)

***ELOCERIA*** Robineau-Desvoidy, 1863

*Eloceria delecta* (Meigen, 1824)

tribe Brachymerini Mesnil, 1939

***PSEUDOPACHYSTYLUM*** Mik, 1891

*Pseudopachystylum gonioides* (Zetterstedt, 1838)

tribe Pelatachinini Mesnil, 1966

***PELATACHINA*** Meade, 1894

*Pelatachina tibialis* (Fallén, 1810)

tribe Macquartiini Robineau-Desvoidy, 1830

***MACQUARTIA*** Robineau-Desvoidy, 1830

*Macquartia dispar* (Fallén, 1820)

*Macquartia nudigena* Mesnil, 1972

= *buccalis* auct. nec Robineau-Desvoidy, 1830

*Macquartia tenebricosa* (Meigen, 1824)

*Macquartia viridana* Robineau-Desvoidy, 1863

***ANTHOMYIOPSIS*** Townsend, 1916

= ***Ptilopsina*** Villeneuve, 1920

*Anthomyiopsis nigrisquamata* (Zetterstedt, 1838)

tribe Triarthriini Belshaw, 1993

***TRIARTHRIA*** Stephens, 1829

*Triarthria setipennis* (Fallén, 1810)

tribe Neaerini Mesnil, 1966

***PHYTOMYPTERA*** Rondani, 1845

= ***Elfia*** Robineau-Desvoidy, 1850

*Phytomyptera bohemica* Kramer, 1907

*Phytomyptera cingulata* (Robineau-Desvoidy, 1830)

*Phytomyptera nigrina* (Meigen, 1824)

*Phytomyptera minutissima* (Zetterstedt, 1844)

*Phytomyptera riedeli* (Villeneuve, 1930)

*Phytomyptera zonella* (Zetterstedt, 1844)

***GRAPHOGASTER*** Rondani, 1868

= ***Anurogyna*** Brauer & Bergenstamm, 1889

*Graphogaster brunnescens* Villeneuve, 1907

*Graphogaster buccata* Herting, 1971

*Graphogaster dispar* (Brauer & Bergenstamm, 1889)

= *punctiventris* (Ringdahl, 1942)

*Graphogaster nigrescens* Herting, 1971

tribe Siphonini Rondani, 1844

***ENTOMOPHAGA*** Lioy, 1864

*Entomophaga nigrohalterata* (Villeneuve, 1921)

*Entomophaga sufferta* (Villeneuve, 1942)

***CEROMYA*** Robineau-Desvoidy, 1830

*Ceromya bicolor* (Meigen, 1824)

*Ceromya dorsigera* Herting, 1967

*Ceromya flaviceps* Ratzeburg, 1844

*Ceromya silacea* (Meigen, 1824)

***ACTIA*** Robineau-Desvoidy, 1830

*Actia crassicornis* (Meigen, 1824)

*Actia infantula* (Zetterstedt, 1844)

*Actia lamia* (Meigen, 1838)

*Actia maksymovi* Mesnil, 1952

*Actia nigroscutellata* Lundbeck, 1927

*Actia pilipennis* (Fallén, 1810)

*Actia resinellae* (Schrank, 1781)

= *nudibasis* Stein, 1924

***PERIBAEA*** Robineau-Desvoidy, 1863

*Peribaea hertingi* Andersen, 1996

= *fissicornis* misid.

*Peribaea longirostris* Andersen, 1996

***CERANTHIA*** Robineau-Desvoidy, 1830

*Ceranthia abdominalis* Robineau-Desvoidy, 1830

*Ceranthia lichtwardtiana* (Villeneuve, 1931)

*Ceranthia pallida* Herting, 1959

*Ceranthia tenuipalpis* (Villeneuve, 1921)

*Ceranthia tristella* Herting, 1966

*Ceranthia verneri* Andersen, 1996

***APHANTORHAPHOPSIS*** Townsend, 1926

*Aphantorhaphopsis samarensis* (Villeneuve, 1921)

*Aphantorhaphopsis siphonoides* (Strobl, 1898)

*Aphantorhaphopsis verralli* (Wainwright, 1928)

***SIPHONA*** Meigen, 1803

*Siphona boreata* Mesnil, 1960

*Siphona collini* Mesnil, 1960

*Siphona confusa* Mesnil, 1961

= *mesnili* Andersen, 1982

*Siphona cristata* (Fabricius, 1805)

= *geniculata* auct. nec (De Geer, 1776)

*Siphona flavifrons* (Staeger, 1849)

*Siphona geniculata* (De Geer, 1776)

= *urbana* (Harris, 1776)

*Siphona grandistyla* Pandellé, 1894

*Siphona immaculata* Andersen, 1996

*Siphona ingerae* Andersen, 1982

*Siphona hokkaidensis* Mesnil, 1957

= *silvarum* Herting, 1967

= *martini* Andersen, 1982

*Siphona maculata* (Staeger, 1849)

*Siphona nigricans* (Villeneuve, 1930)

*Siphona paludosa* Mesnil, 1960

*Siphona pauciseta* Rondani, 1865

*Siphona rossica* Mesnil, 1961

*Siphona setosa* Mesnil, 1960

*Siphona subarctica* Andersen, 1996

*Siphona variata* Andersen, 1982

tribe Leskiini Townsend, 1919

***APHRIA*** Robineau-Desvoidy, 1830

*Aphria longilingua* Rondani, 1861

*Aphria longirostris* (Meigen, 1824)

***DEMOTICUS*** Macquart, 1854

*Demoticus plebejus* (Fallén, 1810)

***LESKIA*** Robineau-Desvoidy, 1830

*Leskia aurea* (Fallén, 1820)

***SOLIERIA*** Robineau-Desvoidy, 1848

*Solieria inanis* (Fallén, 1810)

*Solieria pacifica* (Meigen, 1824)

= *tibialis* (von Roser, 1840)

tribe Minthonini Brauer & Bergenstamm, 1889

***MINTHO*** Robineau-Desvoidy, 1830

*Mintho rufiventris* (Fallén, 1816)

***MINTHODES*** Brauer & Bergenstamm, 1899

*Minthodes picta* (Zetterstedt, 1844)

tribe Microphthalmini Crosskey, 1976

***DEXIOSOMA*** Rondani, 1856

*Dexiosoma caninum* (Fabricius, 1794)

DEXIINAE Macquart, 1834

tribe Dexiini Macquart, 1834

***TRIXA*** Meigen, 1824

= ***Murana*** Meigen, 1824

*Trixa caerulescens* Meigen, 1824

= *alpina* Meigen, 1824

*Trixa conspersa* (Harris, 1776)

= *variegata* Meigen, 1824

= *oestroidea* (Robineau-Desvoidy, 1830)

***BILLAEA*** Robineau-Desvoidy, 1830

*Billaea irrorata* (Meigen, 1826)

*Billaea kolomyetzi* Mesnil, 1970

*Billaea triangulifera* (Zetterstedt, 1844)

***DINERA*** Robineau-Desvoidy, 1830

*Dinera ferina* (Fallén, 1816)

*Dinera grisescens* (Fallén, 1816)

*Dinera carinifrons* (Fallén, 1817)

***ESTHERIA*** Robineau-Desvoidy, 1830

*Estheria petiolata* (Bonsdorff, 1866)

***DEXIA*** Meigen, 1826

*Dexia vacua* (Fallén, 1816)

***PROSENA*** Le Peletier & Serville, 1828

*Prosena siberita* (Fabricius, 1775)

tribe Voriini Townsend, 1912

***ERIOTHRIX*** Meigen, 1803

*Eriothrix argyreatus* (Meigen, 1824)

= *apenninus* misid.

*Eriothrix prolixa* (Meigen, 1824)

*Eriothrix rufomaculata* (De Geer, 1776)

= *monochaeta* (Wainwright, 1928)

***TRAFOIA*** Brauer & Bergenstamm, 1893

*Trafoia monticola* Brauer & Bergenstamm, 1893

***CAMPYLOCHETA*** Rondani, 1859

= ***Elpe*** Robineau-Desvoidy, 1863

*Campylocheta fuscinervis* (Stein, 1924)

*Campylocheta inepta* (Meigen, 1824)

*Campylocheta praecox* (Meigen, 1824)

***BLEPHAROMYIA*** Brauer & Bergenstamm, 1889

*Blepharomyia angustifrons* Herting, 1971

*Blepharomyia pagana* (Meigen, 1824)

= *amplicornis* (Zetterstedt, 1844)

*Blepharomyia piliceps* (Zetterstedt, 1859)

***PETEINA*** Meigen, 1838

*Peteina erinaceus* (Fabricius, 1794)

***RAMONDA*** Robineau-Desvoidy, 1863

*Ramonda prunaria* (Rondani, 1861)

= *carbonaria* misid.

*Ramonda ringdahli* (Villeneuve, 1922)

*Ramonda spathulata* (Fallén, 1820)

***WAGNERIA*** Robineau-Desvoidy, 1830

*Wagneria alpina* (Villeneuve, 1910)

*Wagneria costata* (Fallén, 1820)

***ATHRYCIA*** Robineau-Desvoidy, 1830

= ***Blepharigena*** Rondani, 1856

*Athrycia curvinervis* (Zetterstedt, 1844)

*Athrycia impressa* (van der Wulp, 1869)

*Athrycia trepida* (Meigen, 1824)

*Athrycia* sp. A

***VORIA*** Robineau-Desvoidy, 1830

= ***Plagia*** Meigen, 1838

*Voria ruralis* (Fallén, 1810)

***CYRTOPHLEBA*** Rondani, 1856

*Cyrtophleba ruricola* (Meigen, 1824)

*Cyrtophleba vernalis* (Kramer, 1917)

***KLUGIA*** Robineau-Desvoidy, 1863

*Klugia marginata* (Meigen, 1824)

***CHAETOVORIA*** Villeneuve, 1920

*Chaetovoria antennata* Villeneuve, 1920

***PHYLLOMYA*** Robineau-Desvoidy, 1830

*Phyllomya volvulus* (Fabricius, 1794)

***THELAIRA*** Robineau-Desvoidy, 1830

*Thelaira nigrina* (Fallén, 1817)

= *nigripes* (Fabricius, 1794) preocc.

***HALIDAYA*** Egger, 1856

*Halidaya aurea* Egger, 1856

***STOMINA*** Robineau-Desvoidy, 1830

*Stomina tachinoides* (Fallén, 1816)

tribe Dufouriini Robineau-Desvoidy, 1830

***DUFOURIA*** Robineau-Desvoidy, 1830

*Dufouria chalybeata* (Meigen, 1824)

*Dufouria nigrita* (Fallén, 1810)

***RONDANIA*** Robineau-Desvoidy, 1850

*Rondania dimidiata* (Meigen, 1824)

*Rondania dispar* (Dufour, 1851)

*Rondania fasciata* (Macquart, 1834)

***MICROSOMA*** Macquart, 1855

= ***Campogaster*** Rondani, 1856

*Microsoma exiguum* (Meigen, 1824)

***FRERAEA*** Robineau-Desvoidy, 1830

*Freraea gagatea* Robineau-Desvoidy, 1830

= *albipennis* (Zetterstedt, 1838)

PHASIINAE Robineau-Desvoidy, 1830

tribe Phasiini Robineau-Desvoidy, 1830

***CLYTIOMYA*** Rondani, 1861

*Clytiomya continua* (Panzer, 1798)

***ELIOZETA*** Rondani, 1856

*Eliozeta pellucens* (Fallén, 1820)

***SUBCLYTIA*** Pandellé, 1894

*Subclytia rotundiventris* (Fallén, 1820)

***GYMNOSOMA*** Meigen, 1803

*Gymnosoma clavatum* (Rohdendorf, 1947)

*Gymnosoma dolycoridis* Dupuis, 1961

*Gymnosoma nudifrons* Herting, 1966

***CISTOGASTER*** Latreille, 1804

= ***Pallasia*** Robinaeu-Desvoidy, 1830

*Cistogaster globosa* (Fabricius, 1775)

***PHASIA*** Latreille, 1804

= ***Alophora*** Robineau-Desvoidy, 1830

**sg. *Hyalomya*** Robineau-Desvoidy, 1830

*Phasia pusilla* (Meigen, 1824)

**sg. *Phasia*** Latreille, 1804

*Phasia aurulans* (Meigen, 1824)

*Phasia barbifrons* (Girschner, 1887)

*Phasia hemiptera* (Fabricius, 1794)

*Phasia obesa* (Fabricius, 1798)

*Phasia subcoleoptrata* (Linnaeus, 1767)

tribe Catharosiini Townsend, 1936

***CATHAROSIA*** Rondani, 1868

*Catharosia pygmaea* (Fallén, 1815)

tribe Strongygastrini Townsend, 1936

***STRONGYGASTER*** Macquart, 1834

= ***Tamiclea*** Macquart, 1836

*Strongygaster celer* (Meigen, 1838)

tribe Leucostomatini Townsend, 1908

***LEUCOSTOMA*** Meigen, 1803

*Leucostoma simplex* (Fallén, 1815)

***BRULLAEA*** Robineau-Desvoidy, 1863

*Brullaea ocypteroidea* Robineau-Desvoidy, 1863

***CINOCHIRA*** Zetterstedt, 1845

*Cinochira atra* Zetterstedt, 1945

tribe Cylindromyiini Townsend, 1912

***LOPHOSIA*** Meigen, 1824

**sg. *Lophosia*** Meigen, 1824

*Lophosia fasciata* Meigen, 1824

***CYLINDROMYIA*** Meigen, 1803

**sg. *Cylindromyia*** Meigen, 1803

*Cylindromyia brassicaria* (Fabricius, 1775)

**sg. *Neocyptera*** Townsend, 1916

*Cylindromyia interrupta* (Meigen, 1824)

**sg. *Ocypterula*** Rondani, 1856

*Cylindromyia pusilla* (Meigen, 1824)

***HEMYDA*** Robineau-Desvoidy, 1830

*Hemyda obscuripennis* (Meigen, 1824)

*Hemyda vittata* (Meigen, 1824)

***BESSERIA*** Robineau-Desvoidy, 1830

*Besseria anthophila* (Loew, 1871)

*Besseria melanura* (Meigen, 1824)

***PHANIA*** Meigen, 1824

*Phania curvicauda* (Fallén, 1820)

*Phania funesta* (Meigen, 1824)

= *pseudofunesta* (Villeneuve, 1931)

*Phania thoracica* Meigen, 1824

**OESTRIDAE** Leach, 1815

HYPODERMATINAE Rondani, 1856

***HYPODERMA*** Latreille, 1818

*Hypoderma bovis* (Linnaeus, 1758)

*Hypoderma lineatum* (De Villers, 1789)

*Hypoderma tarandi* (Linnaeus, 1758)

CEPHENEMYIINAE Patton, 1921

tribe Cephenemyiini Patton, 1921

***CEPHENEMYIA*** Latreille, 1818

*Cephenemyia trompe* (Modeer, 1786)

*Cephenemyia ulrichii* Brauer, 1862

GASTEROPHILINAE Girschner, 1896

***GASTEROPHILUS*** Leach, 1817

*Gasterophilus haemorrhoidalis* (Linnaeus, 1758)

*Gasterophilus intestinalis* (De Geer, 1776)

*Gasterophilus nasalis* (Linnaeus, 1758)

## Checklist part 2: Hippoboscoidea

suborder Brachycera Macquart, 1834

clade Eremoneura Lameere, 1906

clade Cyclorrhapha Brauer, 1863

infraorder Schizophora Becher, 1882

clade Muscaria Enderlein, 1936

parvorder Calyptratae Robineau-Desvoidy, 1830

superfamily Hippoboscoidea Samouelle, 1819

**HIPPOBOSCIDAE** Samouelle, 1819

HIPPOBOSCINAE Samouelle, 1819

tribe Ornithomyini Costa, 1846

***CRATAERINA*** von Olfers, 1816

= ***Stenepteryx*** Leach, 1817

*Crataerina hirundinis* (Linnaeus, 1758)

*Crataerina pallida* (Olivier, 1812)

tribe Olfersiini Maa, 1969

***OLFERSIA*** Wiedemann, 1830

= ***Feronia*** Leach, 1817 preocc.

*Olfersia fumipennis* (Sahlberg, 1886)

***ORNITHOMYA*** Latreille, 1802

*Ornithomya avicularia* (Linnaeus, 1758)

*Ornithomya chloropus* Bergroth, 1901

*Ornithomya fringillina* Curtis, 1836

***ORNITHOPHILA*** Rondani, 1879

*Ornithophila metallica* (Schiner, 1864)

tribe Hippoboscini Samouelle, 1819

***HIPPOBOSCA*** Linnaeus, 1758

*Hippobosca equina* Linnaeus, 1758

tribe Lipoptenini Speiser, 1908

***LIPOPTENA*** Nitzsch, 1818

*Lipoptena cervi* (Linnaeus, 1758)

***MELOPHAGUS*** Latreille, 1802

*Melophagus ovinus* (Linnaeus, 1758)

NYCTERIBIINAE Samouelle, 1819

tribe Nycteribiini Samouelle, 1819

***NYCTERIBIA*** Latreille, 1796

**sg. *Nycteribia*** Latreille, 1796

*Nycteribia kolenatii* Theodor & Moscona, 1954

= *pedicularia* auct. nec Latreille, 1805

= *latreillei* misid.

= *blasii* (Kolenati, 1863) preocc.

***PENICILLIDIA*** Kolenati, 1863

*Penicillidia monoceros* Speiser, 1900

## Excluded species

Aphantorhaphopsis (Ceranthia) selecta (Pandellé, 1894) misidentified

*Baumhaueria goniaeformis* (Meigen, 1824) misidentified

*Bithia geniculata* (Zetterstedt, 1844) see Notes

*Blepharipoda scutellata* (Robineau-Desvoidy, 1830) misidentified

*Blondelia piniariae* (Hartig, 1838). Specimens reared from the correct host are mentioned in the notes of Lauri Tiensuu. See Bergström and Bystrowski (2011) for a recent revalidation of this species.

*Billaea fortis* (Rondani, 1862) a likely misidentification, the specimen cannot be located

*Dexia rustica* (Fallén, 1816) misidentified

*Drino atropivora* Robineau-Desvoidy, 1830 not found within present borders

*Entomophaga exoleta* (Meigen, 1824) misidentified

*Graphogaster vestita* Rondani, 1868 misidentified

*Gymnosoma rotundatum* (Linnaeus, 1758) misidentified

*Istocheta subcinerea* (Borisova-Zinoveva, 1966) misidentified

*Leiophora innoxia* (Meigen, 1824) specimen lost, but likely a misidentification due to the host record (*Diprion* sp. Symphyta: Diprionidae)

*Linnaemya comta* (Fallén, 1810) misidentified

*Lydella grisescens* Robineau-Desvoidy, 1830 misidentified

*Macronychia alpestris* Rondani, 1865 labeling mistake

*Macronychia conica* Robineau-Desvoidy, 1830 labeling mistake

*Macroprosopa atrata* (Fallén, 1810) misidentified

*Masicera silvatica* (Fallén, 1810) misidentified

*Oestrus ovis* Linnaeus, 1758 extinct?

*Opesia cana* (Meigen, 1824) not found within present borders

*Pseudogonia rufifrons* (Wiedemann, 1830) imported

*Pseudoperichaeta palesoidea* (Robineau-Desvoidy, 1830) misidentified

*Sarcophaga agnata* Rondani, 1860 misidentified

*Sarcophaga dissimilis* Meigen, 1826 misidentified

*Sarcophaga granulata* Kramer, 1908

*Sarcophila latifrons* (Fallén, 1817) not found within present borders

*Senometopia confundens* Rondani, 1859 misidentified

*Thelymorpha marmorata* (Fabricius, 1805), record cannot be confirmed – specimen lost?

## Notes

***Athrycia* sp. A**. Probably an undescribed species (J. Flinck leg., T. Zeegers priv. comm.)

***Bithia geniculata* (Zetterstedt, 1844)**. Erroneously included in the previous checklist (Hackman 1980, 1981) on the basis of misintepreted records of *Siphona geniculata* (De Geer, 1776).

***Dexia vacua* (Fallén, 1816)** and ***Dinera carinifrons* (Fallén, 1817)**. Conspiguous large species with many historical records, however they have not been seen for decades in Finland (last record for *Dinera carinifrons* from 1945). While the species have certainly become rarer, their complete extinction cannot be confirmed due to the regional paucity of recording. For example, *Linnaemya rossica* Zimin, 1954 was similarly missing for 50 years until rediscovered in 2013 from Lieksa, eastern Finland.

***Exorista* sp. A**. Probably an undescribed species, near to *Exorista tubulosa* Herting, 1967 (C. Bergström, pers. comm.)

***Linnaemya* sp. A**. An undescribed species (C. Bergström, pers. comm.). A highly unusual, shiny black *Linneamya* species from Lapland.

***Nemorilla floralis* (Fallen, 1810)**. *Nemorilla maculosa* (Meigen, 1824) has been mixed with this species on Hackman´s (1980) list. True *floralis* was found new to Finland in 2011 from Loviisa (J. Flinck leg.).

***Phebellia* sp. A**. Probably an undescribed species (J. Flinck leg., T. Zeegers priv. comm.)

***Stomorhina lunata* (Fabricius, 1805)**. Recorded once from the University Botanical Gardens in Helsinki (2 exx). It is quite possible that the flies were imported to Finland, but the species is also a known vagrant and the possibility of natural dispersal cannot be excluded.

**Tachina sp. aff. magnicornis**. Close to the real *Tachina magnicornis* of Zetterstedt. The proper name of this species still under investigation, but may be *Tachina tetramera* (Zetterstedt, 1849). It is common and widespread in Finland. Some records have been confirmed by the DNA barcoding project (Pohjoismäki et al. 2013). See Tschorsnig et al. (2002) and Novotna et al. (2009).

## References

[B1] Bergström C Bystrowski C (2011) The identity of *Blondelia pinivorae* (Ratzeburg) (Diptera: Tachinidae), a parasitoid of processionary moths (Lepidoptera: Thaumetopoeidae). Stuttgarter Beiträge zur Naturkunde A, Neue Serie 4: 321–334

[B2] Dick CW (2006) Checklist of world Hippoboscidae (Diptera: Hippoboscidae). Department of Zoology, Field Museum Natural History, Chicago, IL

[B3] Hackman W (1980) A Check List of the Finnish Diptera. Notulae entomologicae 60: 17–48, 117–162

[B4] Hackman W (1981) För Finland nya flugor samt tre övriga till ägg till förtäckningen över Finlands Diptera. Notulae entomologicae 61: 225–226

[B5] Herting B (1993) Family Rhinophoridae. In: Soós Á Papp L (Eds) Catalogue of Palaearctic Diptera 13. Hungarian Natural History Museum, Budapest, 102–117

[B6] Herting B Dely-Draskovits A (1993) Family Tachinidae. In: Soós Á Papp L (Eds) Catalogue of Palaearctic Diptera 13. Hungarian Natural History Museum, Budapest, 118–458

[B7] Kutty SN Pape T Wiegmann BM Meier R (2010) Molecular phylogeny of the Calyptratae (Diptera: Cyclorrhapha) with an emphasis on the superfamily Oestroidea and the position of Mystacinobiidae and McAlpine’s fly. Systematic Entomology 35: 614–635. doi: 10.1111/j.1365-3113.2010.00536.x

[B8] Marinho MA Junqueira AC Paulo DF Esposito MC Villet MH Azeredo-Espin AM (2012) Molecular phylogenetics of Oestroidea (Diptera: Calyptratae) with emphasis on Calliphoridae: insights into the inter-familial relationships and additional evidence for paraphyly among blowflies. Molecular Phylogenetics and Evolution 65: 840–754. doi: 10.1016/j.ympev.2012.08.007 22926310 10.1016/j.ympev.2012.08.007

[B9] Novotná H Vanhara J Tóthová A Muráriková N Bejdák P Tkoč M Rozkošný R (2009) Identification and taxonomy of the West Palaearctic species of *Tachina* Meigen (Diptera: Tachinidae) based on male terminalia and molecular analyses. Entomologica Fennica 20(3): 139–169

[B10] O’Hara JE (2012) World genera of the Tachinidae (Diptera) and their regional occurrence. Version 7.0. PDF document, 75 pp. http://www.nadsdiptera.org/Tach/WorldTachs/Genera/Gentach_ver7.pdf [accessed Feb 6, 2014]

[B11] Pape T (1987a) An annotated check-list of Finnish flesh-flies (Diptera: Sarcophagidae). Notulae Entomologicae 67: 43–46

[B12] Pape T (1987b) The Sarcophagidae (Diptera) of Fennoscandia and Denmark. Fauna Entomologica Scandinavica 19. Scandinavian Science Press Ltd., Leiden-New York-København-Köln, 203 pp

[B13] Pape T (1996) Catalogue of the Sarcophagidae of the World (Insecta: Diptera). Memoirs on Entomology, International American Entomological Institute, Gainsville, 558 pp

[B14] Pape T (2001) Phylogeny of Oestridae (Insecta: Diptera). Systematic Entomology 26(2): 133–171. doi: 10.1046/j.1365-3113.2001.00143.x

[B15] Pape T Blagoderov V Mostovski MB (2011) Order Diptera Linnaeus, 1758. In: Zhang Z-Q (Ed) Animal biodiversity: An outline of higher-level classification and survey of taxonomic richness.Zootaxa 3148: 222–229 10.11646/zootaxa.3703.1.1 26146682

[B16] Pohjoismäki JL Kahanpää J Mutanen M (2013) DNA barcodes for north European Tachinidae: preliminary results and material request. Tachinid Times 26: 17–19

[B17] Rognes K (1991) Blowflies (Diptera, Calliphoridae) of Fennoscandia and Denmark. Fauna Entomologica Scandinavica 24. Scandinavian Science Press Ltd., Leiden-New York-København-Köln, 272 pp

[B18] Tschorsnig H-P Ziegler J Herting B (2002) Tachinid flies (Diptera: Tachinidae) from the Hautes-Alpes, France. Stuttgarter Beiträge zur Naturkunde, Serie A (Biologie) 656: 1–62

